# Metal and Metalloid-Induced Oxidative Damage: Biological Importance of Potential Antioxidants

**DOI:** 10.1155/2018/3586071

**Published:** 2018-07-25

**Authors:** Maria Fernanda Hornos Carneiro, Gustavo Rafael Mazzaron Barcelos, Fernando Barbosa, Joseph Adeyemi, Glenda Gobe

**Affiliations:** ^1^Department of Clinical Analysis, Toxicology and Food Science, School of Pharmaceutical Sciences of Ribeirao Preto, University of Sao Paulo, Ribeirão Preto, SP, Brazil; ^2^Department of Biosciences, Institute of Health and Society, Federal University of Sao Paulo, Santos, SP, Brazil; ^3^Department of Biology, School of Sciences, Federal University of Technology, Akure, Ondo State, Nigeria; ^4^Kidney Disease Research Collaborative, Translational Research Institute, School of Biomedical Sciences, Faculty of Medicine, NHMRC CKD.QLD CRE, Royal Brisbane and Women's Hospital, University of Queensland, Brisbane, QLD, Australia

A general disregard by industries and big corporations for the environment has meant that the natural environment has repeatedly been polluted, jeopardizing the well-being of all living organisms. The toxic effects of metals and metalloids have been recognized for quite some time to generate several free radicals and other reactive species that lead to cellular oxidative stress. Metals and metalloids are toxic elements at the top of the priority list of hazardous substances of the Agency for Toxic Substances and Disease Registry (ATSDR). Gaps of knowledge still exist that are related to their toxicity, mainly concerning the mechanisms of action. Due to their extreme potential to cause oxidative damage, the structural, functional, biochemical, and molecular effects of metals and metalloids on oxidative stress need description, and the identification of new potential competitive-protective agents is imperative. This special issue brings together the results of six papers covering several aspects of the toxicology of metals and metalloids in *in vitro* and *in vivo* experimental models. They provide a deeper understanding of the mechanisms involved in metal and metalloid toxicity and highlight the need to develop strategies to decrease exposure to metals, as well as identify substances that may help overcome the hazardous effects within the living body.

Carnosic acid, a natural benzenediol abietane diterpene found in rosemary (*Rosmarinus officinalis),* was explored to rescue the arsenite-induced oxidative stress in hepatic cells in *in vitro* and *in vivo* models in the study of S. Das et al. Carnosic acid was able to significantly rescue the activation of MAPK, NF-*κ*B, p53, and intrinsic and extrinsic apoptotic signaling seen in arsenite-exposed hepatic cells. Carnosic acid could also significantly counteract with redox stress and ROS-mediated signaling, thereby attenuating arsenite-mediated hepatotoxicity. Other effects, such as arsenic bioaccumulation, DNA fragmentation, and hematological changes, were also reverted in models after coadministration with carnosic acid. The authors attributed the rescue to the antioxidant potential of carnosic acid which in their view had the potential to be a new therapeutic agent to counteract with arsenite-mediated toxic manifestations. In the same way, the study of A. Officioso et al. compared the beneficial effects of olive oil phenol hydroxytyrosol (HT) and 5-*S*-lipoylhydroxytyrosol (Lipo-HT) on oxidative alterations of human erythrocytes induced by exposure to inorganic mercury. Both HT and Lipo-HT decreased Hg-induced generation of ROS, hemolysis, and the depletion of intracellular GSH levels. At all doses tested, Lipo-HT exhibited a higher ability to counteract Hg-induced cytotoxicity compared to HT. To explain that, the authors propose a model where Lipo-HT is more effective to chelate mercury ions and encourage the use of Lipo-HT in nutraceutical strategies to counteract heavy metal toxicity in humans.

Obesity- or diabetes-induced oxidative stress is discussed as a major risk factor for DNA damage. What has not been addressed yet is how vitamin E and many polyphenols exhibit antioxidative activities with consequences on epigenetic regulation of inflammation and DNA repair. To shed light on that, the study of K. Zappe et al. investigated the counteraction of oxidative stress by vitamin E in the colorectal cancer cell line Caco-2 under normal- and high-glucose cell culture condition. Results revealed a dose-dependent counteracting effect of vitamin E on malondialdehyde levels. Also, an induction in the expression of the genes of DNA repair *MutL homolog (MLH1)* and the *DNA methyltransferase 1 (DNMT1)* was noticed, accompanied by an increase in global methylation by LINE-1. The authors suggest that vitamin E supplementation has a high potential for treatment and might even be used as a possible approach in the prevention of diseases caused by obesity and diabetes.

Cadmium is one of the most poisonous environmental chemicals, causing toxicity in humans and experimental animals. The study of M. C. Cupertino et al. investigated the relationship between germ and Leydig cell death, testosterone, and adiponectin levels in cadmium-mediated acute toxicity. Dose-dependent cadmium accumulation in testes was identified. At the highest doses of exposure, animals exhibited marked inflammatory infiltrate and disorganization of the seminiferous epithelium. Although Leydig cells were morphologically resistant to Cd toxicity, massive germ cell death, DNA oxidation, and fragmentation were observed. Also, testosterone and adiponectin levels were significantly decreased by cadmium. The authors discuss the mechanisms behind the findings, but why germ cells are a primary target of cadmium-induced toxicity while Leydig cells remain resistant to death even when exposed to higher doses of this metal is still unclear.

Despite the undoubted advantages of titanium bone fixations such as high biotolerance, favorable mechanical properties, and osseointegration ability, their negative impact on the human body, both at the implant site and at the systemic level, is still debated. To contribute to the toxicological background in this emergent area, the study of J. Borys et al. evaluated the influence of Ti6Al4V titanium alloy on redox balance and oxidative damage in the periosteum surrounding the titanium miniplates and screws as well as in plasma and erythrocytes of patients with mandibular fractures. The occurrence of a redox imbalance as well as oxidative damage in the periosteum surrounding the Ti6Al4V titanium alloy was demonstrated. Changes in antioxidant potential in the patients treated with titanium implants were also observed in the plasma/erythrocytes. Results indicate the need to improve the miniplates and screws used for osteosynthesis by increasing the thickness of the passive TiO_2_ layer or using new, biodegradable, and more biocompatible materials. The authors also suggest that supplementation with antioxidants could be tested as a therapeutic procedure in patients treated with titanium fixations.

Finally, S. R. Lee critically reviewed the role of zinc as either an antioxidant or a prooxidant. For instance, it functions as an antioxidant through the catalytic action of copper/zinc-superoxide dismutase, stabilization of membrane structure, protection of the protein sulfhydryl groups, and upregulation of the expression of metallothionein, which possesses a metal-binding capacity and exhibits antioxidant functions. Also, zinc can suppress anti-inflammatory responses that would otherwise augment oxidative stress. On the other hand, zinc deficiency and zinc excess cause cellular oxidative stress. The conditions behind the dual actions of zinc and the oxidative imbalance that occurs in zinc deficiency and zinc overload in conjunction with the intracellular regulation of free zinc are summarized in this review.

We hope that the new findings on the toxicity of metals and metalloids as well as on the identification of protective agents presented in this special edition can contribute to the development of science, to the environmental and health regulatory agency policies, and to more effective strategies to rescue toxicity.

